# Airflow Features Obtained From Voluntary Throat Clearing Compared to Voluntary Cough and Induced Reflexive Cough in a Healthy Population

**DOI:** 10.1111/1460-6984.70160

**Published:** 2025-11-17

**Authors:** Sofiana Mootassim‐Billah, Gwen Van Nuffelen, Jean Schoentgen, Marc De Bodt, Hichem Slama, Mathilde Le Tensorer, Dirk Van Gestel

**Affiliations:** ^1^ Department of Radiation Oncology, Speech Therapy Unit, Institut Jules Bordet Hôpital Universitaire de Bruxelles, Université Libre de Bruxelles Brussels Belgium; ^2^ Department of Otolaryngology and Head and Neck Surgery University Rehabilitation Center for Communication Disorders, Antwerp University Hospital Antwerp Belgium; ^3^ Faculty of Medicine and Health Sciences Department of Translational Neurosciences University of Antwerp Antwerp Belgium; ^4^ Faculty of Medicine and Health Sciences Department of Logopaedics and Audiological Sciences, University of Ghent Ghent Belgium; ^5^ Department of Biomechatronics Université Libre de Bruxelles Brussels Belgium; ^6^ Department of Neuropsychology and Speech Therapy, CUB Hôpital Erasme Hôpital Universitaire de Bruxelles, Université libre de Bruxelles Brussels Belgium; ^7^ UR2NF, Neuropsychology and Functional Neuroimaging Research Unit at CRCN Centre de Recherches en Cognition et Neurosciences Brussels Belgium

**Keywords:** airflow features, airway protective manoeuvres, coughing, dysphagia, throat clearing

## Abstract

**Background:**

Coughing and throat clearing are different airway protective manoeuvres elicited in the framework of dysphagia. However, coughing and throat clearing may be auditorily confused during a clinical swallowing evaluation. In addition, literature reporting comparisons between coughing and throat clearing via gold standard airflow metrics is lacking.

**Aims:**

To report quantitative airflow data for voluntary throat clearing, and to examine in a healthy population the aerodynamical differences between voluntary throat clearing, voluntary cough and induced reflexive cough.

**Methods and Procedures:**

Forty healthy participants were included in the study. Airflow measurements were obtained from single voluntary throat clearings, single voluntary coughs and the first two induced reflexive coughs of the reflexive cough epoch. The measurements included the peak expiratory flow rate in litres/s and the cough expired volume in litres of each single manoeuvre.

**Outcomes and Results:**

Results showed that voluntary throat clearing displayed lower airflow feature values compared to voluntary cough and induced reflexive cough (*p* < 0.001).

**Conclusions and Implications:**

Voluntary throat clearings were aerodynamically significantly different from voluntary and reflexive coughs. Future studies should determine whether these differences reflect distinct impacts on airway protection. In dysphagic populations, instrumental assessment of throat clearing may enhance clinical swallowing evaluations.

**WHAT THIS PAPER ADDS:**

*What is already known on this subject*

- A large body of literature has emphasized airflow differences between voluntary coughs and induced reflexive coughs, supporting physiological differences between both types of manoeuvres and complementary roles with regard to airway protection. Throat clearing is another airway protective manoeuvre anecdotally considered during a clinical swallowing evaluation and in literature. In addition, throat clearing is confused with coughing, and little is known regarding its ability to protect the airways.

*What this study adds to existing knowledge*

- This exploratory study is the first to report quantitative airflow metrics for voluntary throat clearings, and to demonstrate significantly lower airflow features for voluntary throat clearing compared to voluntary cough and induced reflexive cough, in a healthy population. The combination of airflow features obtained in this study with anatomical observations and acoustical features increases insight into the physiological differences between throat clearing and coughing.

*What are the potential or actual clinical implications of this work?*

- The study demonstrates that the instrumental assessment of throat clearing as a distinctive airway protective manoeuvre would be of value to enable more accurate clinical swallowing evaluation and management of dysphagic patients. Further investigation and consideration of throat clearing are warranted.

## 1 Introduction

Cough is a protective sensorimotor behaviour the function of which is to eject materials (food or secretions) from the airways. In this protective function, cough prevents life‐threatening complications such as aspiration pneumonia in patients with dysphagia at risk of penetration/aspiration. Indeed, the co‐occurrence of coughing and swallowing dysfunction has been reported to enhance the aspiration risk in different populations with dysphagia (Hammond et al. 2001; Lee et al. 2015; Nguyen et al. 2007; Pitts et al. 2008; Plowman et al. 2016; Troche et al. 2014, 2016).

In the framework of dysphagia, cough airflow‐related measures are regarded as the established instrumental cough assessment. The peak expiratory flow rate (PEFR) in litres/second (L/s) and the cough expired volume (CEV) in litres (L) have been reported to be the most reliable airflow parameters that predict the risk of penetration/aspiration (Curtis and Troche 2020; Plowman et al. 2016; Troche et al. 2016). The PEFR is the peak airflow rate in the expiratory phase that depends on: (a) glottal compression (vocal fold adduction) enabling sufficient subglottic pressure before reopening of the glottis to result in a forced expiratory effort; (b) the operating volume, that is, the air volume inspired prior to coughing (Fullerton et al. 2021; Smith et al. 2012). The CEV is the total volume of air expired from the beginning of the expiratory phase of the first cough to the last cough of the cough epoch. PEFR and CEV are positively correlated (Hegland et al. 2013) because they both are influenced by the amount of air in the lungs at cough initiation (Smith et al. 2012), the total number of coughs in a cough epoch (Hegland et al. 2013), and the coordination between laryngeal and respiratory subsystems (Poliaček et al. 2003).

Aerodynamic cough assessment involves the measurement of PEFR and CEV obtained from both voluntary and induced reflexive coughs. Induced reflexive coughs are obtained by means of an inhalation cough challenge with tussive agents such as capsaicin or citric acid, enabling cough induction in a dose‐dependent and reproducible manner (Morice et al. 2007; Wallace et al. 2019). It is widely reported that voluntary coughs and induced reflexive coughs are aerodynamically distinguishable, suggesting physiological differences and complementary roles with regard to airway protection (Borders and Troche 2022; Mills et al. 2017; Pitts et al. 2008; Tabor‐Gray et al. 2019; Troche et al. 2016).

Throat clearing is another behaviour that is part of the continuum of airway protective manoeuvres (Troche et al. 2014; Vertigan and Gibson 2016). However, its consideration during a clinical swallowing evaluation is variable across clinical departments and depends on clinicians’ habits. Similarly to coughing, throat clearing may be produced in the case of entry of materials in the laryngeal vestibule. Physiologically speaking, the difference between throat clearing and coughing is that the former does not have a mandatory inspiratory phase and does not require complete glottal closure (Laciuga et al. 2016; Xiao et al. 2014). Acoustically speaking, throat clearing is distinguishable from coughing because it is characterized by one main signal fragment involving acoustical oscillations all along (Mootassim‐Billah et al. 2023). In contrast, a cough signal involves two or three signal fragments, including a strong onset‐pulse followed by frication noise and a non‐mandatory voiced coda characterized by acoustical oscillations (Mootassim‐Billah et al. 2023). Despite significant acoustical differences (Mootassim‐Billah et al. 2023) and training in auditory‐perceptual assessment (Curtis et al. 2024), clinicians are poor at discriminating coughing and throat clearing auditorily (Laciuga et al. 2016). Also, literature reporting comparisons between coughing and throat clearing via gold standard airflow metrics is lacking. Therefore, little is known regarding the actual ability of throat clearing to protect and clear the airways.

The aims of this article are to report quantitative airflow data for voluntary throat clearings, and to compare airflow features between voluntary throat clearings and voluntary coughs as well as induced reflexive coughs in a healthy population. The comparison of airflow features aims at distinguishing between the aerodynamic characteristics of each manoeuvre in a healthy population, which may inform future research into their potential roles in airway clearance. The study we report is part of overall exploratory research aiming at detecting dysphagic patients showing impaired airway protective manoeuvres, based on aerodynamic and acoustic features, and exploring the place of the throat clearing manoeuvre in the continuum of airway protective manoeuvres.

## 2 Methods

### 2.1 Participants

Forty healthy adult individuals participated in this study (> 18 years). Inclusion criteria were: (1) lack of relevant medical complaints; (2) lack of cognitive complaints; (3) no history of head and neck cancer; (4) no dysphagia (according to the Yale Swallow Protocol; Suiter et al. 2014); (5) no dysphonia (*G* = 0 on the GRBAS‐I scale; Hirano 1981); (6) no history of smoking within less than 1 year; (7) no acute or chronic respiratory disease (e.g., chronic obstructive pulmonary disease or asthma). Participants were recruited on a voluntary basis among hospital staff and external hospital parties.

### 2.2 Airflow Measurements

Airflow measurements were obtained from five single voluntary coughs, five single voluntary throat clearings and the two first induced reflexive coughs of the reflexive cough epoch (C2 threshold). The measurements included the PEFR in L/s and the CEV in litres. These features were selected as primary airflow measures in this exploratory study due to their frequent use in previous cough studies and their suitability for the present equipment setup.

### 2.3 Instructions to Participants

Participants were seated upright throughout all manoeuvres to ensure consistency of posture and measurement conditions. For voluntary coughing, each participant was verbally instructed (in French) as follows: ‘Take a maximal breath and cough once as if something is stuck in your throat.’For voluntary throat clearing, the verbal instruction was ‘Take a breath and clear your throat as if something is stuck in your throat.’Participants producing a voluntary cough rather than a throat clearing following this instruction were given a demonstration by the experimenter. For cough induction only, participants were divided into two groups. The first group (*N* = 20) was instructed to ‘try not to cough’ (the suppressed reflexive cough method, SRC). The second group (*N* = 20) was initially instructed to ‘breathe through your mouth and cough if you need to’ (the urge‐to‐cough method, UTC). After recording 2 successive coughs according to the UTC method, this group received incremental citric acid concentrations from the cough threshold, now with the instruction ‘breathe through your mouth and try not to cough.’ This enabled recording induced reflexive coughs via two different methods.

### 2.4 Equipment

Cough airflow waveforms were recorded via an anaesthesia facemask connected to a spirometer, Pocket‐Spiro USB100 (Medical Electronic Construction; https://mecrd.eu/), with a scale ranging from 0.05 to 15 L/s ± 2%. Calibration of the spirometer had to be performed daily to ensure the quality of measurements, according to the manufacturer's protocol. Cough airflow waveforms were digitized and stored on a desktop computer via PDI software version 2021 (Medical Electronic Construction; https://mecrd.eu/).

Induced reflexive coughs were recorded using a facemask covering the mouth and nose, coupled to a Pocket‐Spiro USB (Medical Electronic Construction; https://mecrd.eu/) and a differential pressure transducer, with a one‐way inspiratory valve for nebulizer connection. The citric acid aerosol was delivered using a Challenger 100 nebulizer system (Medical Electronic Construction; https://mecrd.eu/), which includes an integrated compressor delivering up to 8 L/s at 1.5 bar and automatic breath recognition via a piezo pressure sensor. The nebulizer was a SIDESTREAM model producing aerosol with 85% of particles smaller than 5 µm and a mass median aerodynamic diameter (MMAD) of 3.75 µm. Nebulization was controlled to occur during a 2‐s deep inspiration (adjustable between 0.1 and 9.9 s from inspiration onset). Citric acid solutions were freshly prepared on the day of testing, diluted in sterile 0.9% saline without pH adjustment. Each participant completed a maximum of 5 challenges with increasing citric acid concentrations: 0 mM (saline), 30 mM (5.8 mg/mL), 100 mM (19.2 mg/mL), 300 mM (58 mg/mL), and 1000 mM (192 mg/mL), following the protocol described in Janssens et al. (2015). A 1‐min latency was maintained between trials to reduce tachyphylaxis (desensitization effects). The challenge protocol was stopped when the C2 threshold—defined as two or more successive coughs triggered after a single inspiration—was reached.

### 2.5 Statistics

A statistical software package (IBM SPSS Statistics 29) was used to obtain descriptive statistics of voluntary throat clearings, voluntary coughs and induced reflexive coughs. Separate analyses were conducted to compare (1) voluntary throat clearings with voluntary coughs, (2) voluntary throat clearings with induced reflexive coughs, and (3) voluntary coughs with induced reflexive coughs. No statistical comparison of CEV was carried out between voluntary throat clearings and induced reflexive coughs as well as between voluntary coughs and induced reflexive coughs because participants were instructed to cough once only for voluntary throat clearings and voluntary coughs, but no specific instruction was given for induced reflexive coughs.

Because this study is part of an exploratory research project, non‐parametrical Wilcoxon and Mann–Whitney *U* tests were used for comparisons between protective manoeuvres. A *p* value of < 0.05 was considered to be statistically significant.

## 3 Results

Forty healthy individuals, including 25 women and 15 men, participated in this study. The average age of the participants was 40.1 ± 10.3 years (range, 24 to 65 years). The average age for the women was 39.5 ± 11.1 (range, 24 to 59), and the average age for the male subjects was 41.1 ± 12.5 (range, 29 to 65).

The median of the PEFR of the voluntary coughs was 3.75 L/s, which is in line with literature reporting that normal PEFR is higher than 3 L/s in healthy individuals (Bach and Zhitnikov 1998; Cardoso et al. 2012; Gauld and Boynton 2005; Suleman et al. 2004; Tabor‐Gray et al. 2020). Anthropometric data such as height, weight and body mass index (BMI) were retrospectively collected in 35/40 (87.5%) of the participants. Demographic and anthropometric data are reported in Table 1.

**TABLE 1:** Demographic and anthropometric data of participants.

Age (y)	*N* = 40	Mean ± SD	40.10 ± 10.30
		Range	24–65
Gender	*N* = 40	Females	*N* = 25 (62.5%)
		Males	*N* = 15 (37.5%)
Height (m)	*N* = 35	Mean ± SD	1.71 ± 0.09
		Range	1.53–1.89
Weight (kg)	*N* = 35	Mean ± SD	74.49 ± 15.41
		Range	46–105
Body mass index (kg/m^2^)	*N* = 35	Mean ± SD	25.37 ± 5.07
		Range	18.90–36.44

All samples of voluntary coughs (*N* = 200) were considered suitable for analysis. Fifteen samples of throat clearings were excluded because participants produced double throat clearings after one inspiration instead of one (total number of throat clearings analysed *N* = 185). As preliminary analyses showed no significant differences in PEFR and CEV between UTC and suppressed reflexive coughs (*p* > 0.05, with overlapping confidence intervals for the medians), all induced reflexive coughs were pooled for analysis (total number of single induced reflexive coughs: *N* = 98).

The medians, quartiles, minima, maxima, and bootstrapped confidence intervals for the medians (95%) of the airflow features collected were obtained (Table 2), and statistical comparisons by means of non‐parametric Wilcoxon and Mann–Whitney *U* tests (with statistically significant difference at *p* < 0.05) were carried out between airway protective manoeuvres.

1. Voluntary throat clearings versus voluntary coughs

**TABLE 2:** Medians, quartiles, minima, maxima, and the confidence intervals (95%) for the medians of the peak expiratory flow rate and the total cough expired volume of voluntary coughs, voluntary throat clearings and induced reflexive coughs.

	Voluntary coughs (*N* = 200)						Voluntary throat clearings (*N* = 185)						Induced reflexive coughs (*N* = 98)					
	Min	Q1	Med	Q3	Max	CI 95% of med	Min	Q1	Med	Q3	Max	CI 95% of med	Min	Q1	Med	Q3	Max	CI 95% of med
PEFR(L/s)	0.188	3.000	3.775	4.645	7.740	3.560–4.010	0.440	1.060	1.590	2.370	6.870	1.450–1.820	0.390	1.735	2.470	3.153	5.310	2.050–2.755
CEV (L)	0.190	0.740	1.010	1.468	3.030	0.915–1.060	0.110	0.345	0.580	0.950	2.800	0.500–0.660	0.320	0.898	1.220	1.538	4.120	1.080–1.320

**FIGURE 1:**
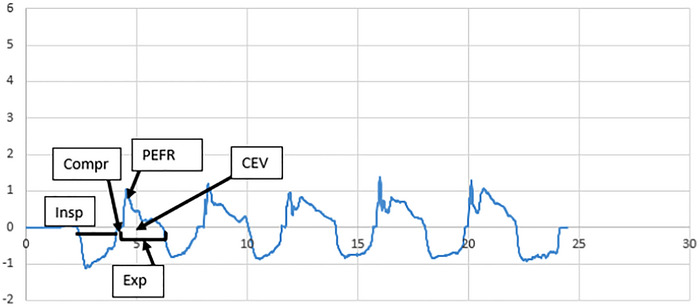
Example of voluntary throat clearing airflow waveform. x‐axis: time (s); y‐axis: flow rate (L/s). CEV = cough expired volume over the expiration phase (area under the curve during this phase), Compr = compression phase, Exp = expiration phase, Insp = inspiratory phase, PEFR = peak expiratory airflow.

**FIGURE 2:**
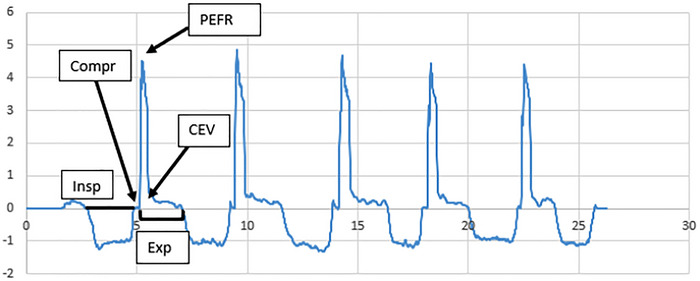
Example of voluntary cough airflow waveform*. x‐*axis: time (s); *y‐*axis: flow rate (L/s). CEV = cough expired volume over the expiration phase (area under the curve during this phase), Compr = compression phase, Exp = expiration phase, Insp = inspiratory phase, PEFR = peak expiratory airflow.

The PEFR and the total expired volume were significantly lower for voluntary throat clearings compared to voluntary coughs (*p* < 0.001) (Table 3). The confidence intervals of the medians did not overlap (Table 2). The most prominent visual difference was low multiple‐peak flow rates for throat clearings and high signal‐peak flow rates for voluntary coughs (Figures 1 and 2).

**TABLE 3:** Non‐parametric comparisons between airway protective manoeuvres and associated *p* values.

	Voluntary throat clearings versus voluntary coughs (Wilcoxon test)	Voluntary throat clearings versus induced reflexive coughs (Mann–Whitney U test)	Voluntary coughs versus induced reflexive coughs (Mann–Whitney *U* test)
PEFR (L/s)	*p* < 0.001	*p* < 0.001	*p* < 0.001
CEV (L)	*p* < 0.001	Not calculated*	Not calculated*

*Given the variety in the number of coughs produced by the participants (Section 2, Methods: Statistics).

2. Voluntary throat clearings versus induced reflexive coughs

**FIGURE 3:**
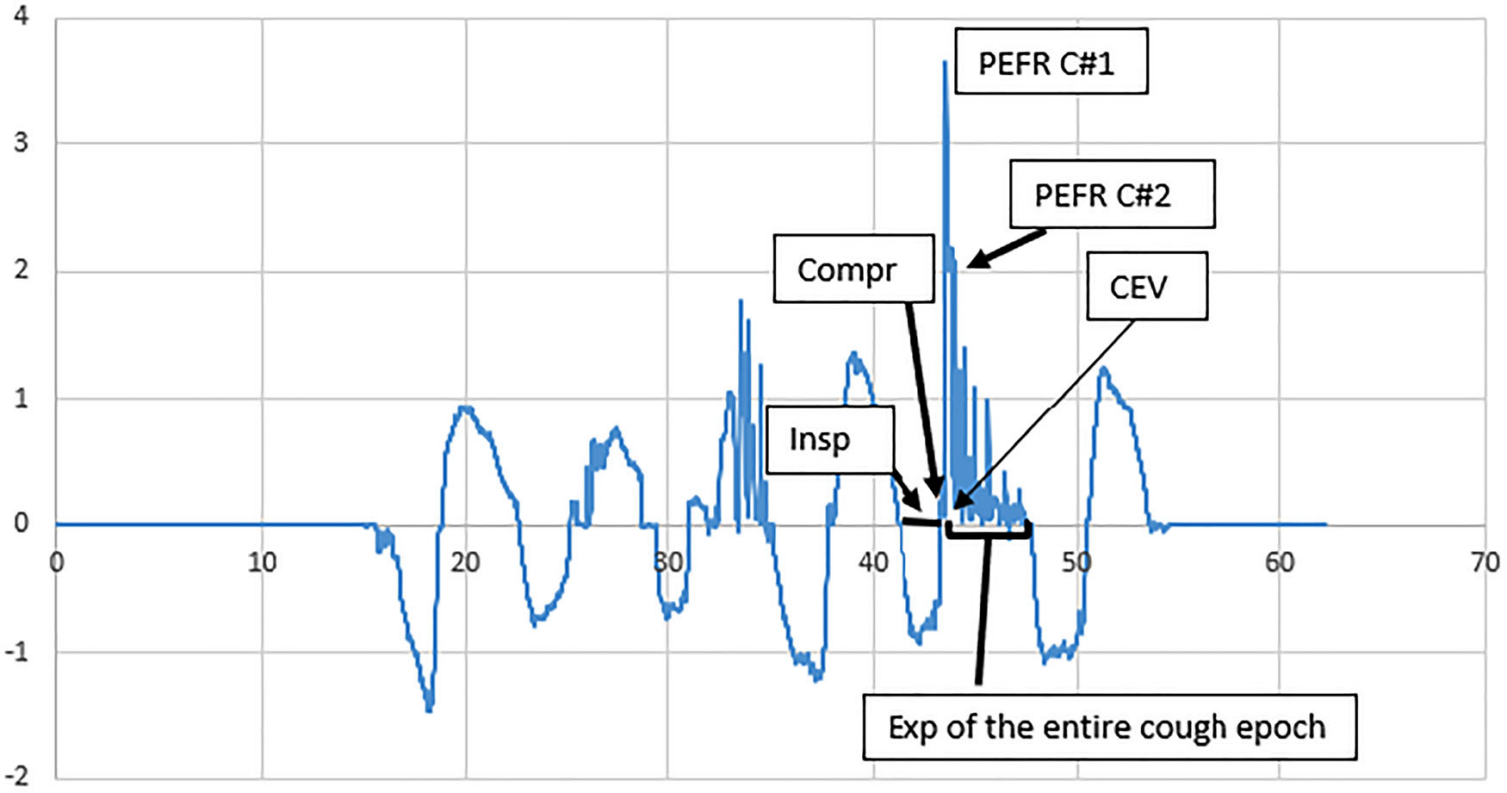
Example of induced reflexive cough airflow waveform*. x‐*axis: Time (s); *y‐*axis: flow rate (L/s). CEV = cough expired volume over the expiration phase of the entire cough epoch (area under the curve during this phase), Compr = compression phase, Insp = inspiratory phase, Exp = expiration phase of the entire cough epoch (the totality of coughs produced after one inspiration), PEFR C#1 = peak expiratory airflow of the first cough of the cough epoch, PEFR C#2 = peak expiratory airflow of the second cough of the cough epoch.

The PEFR was significantly lower for voluntary throat clearings compared to induced reflexive coughs (*p* < 0.001, no overlap of the confidence intervals for the medians) (Tables 2 and 3). A visual inspection showed that induced reflexive coughs started with a high peak flow rate followed by subsequent lower peaks (Figure 3).

3. Voluntary coughs versus induced reflexive coughs

The PEFR was significantly higher (*p* < 0.001, no overlap of the confidence intervals for the medians) for voluntary coughs compared to induced reflexive coughs (Tables 2 and 3). A visual inspection shows that cough airflow waveforms of the induced reflexive cough epoch were characterized by a rapid decrease of the PEFR from the second cough on (Figures 2 and 3).

## 4 Discussion

The aim of this study was to report quantitative airflow data for voluntary throat clearings, and to compare the aerodynamic features of voluntary throat clearings, voluntary coughs, and induced reflexive coughs in a healthy population. This comparison may help guide future research into the distinct roles these manoeuvres may play in airway protection and clearance. This study is part of an overall research project with a view to detecting, based on aerodynamic and acoustic features, dysphagic patients with impaired protective manoeuvres. Ultimately, we expect that the combined recording of aerodynamic and acoustic features as potential biomarkers of dysphagia will extend our knowledge of the physiology of these distinct airway protective manoeuvres.

Comparisons of throat clearing with coughing via airflow metrics are lacking. Also, throat clearing may be auditorily confused with coughing, even though throat clearing is a clearance manoeuvre occasionally assessed during a clinical swallowing examination. Therefore, we analysed the differences between throat clearing and coughing by examining the PEFR and the CEV that are reported to be the most reliable predictors of the penetration/aspiration risk (Curtis and Troche 2020; Plowman et al. 2016; Troche et al. 2016). To our knowledge, this study is the first that reports quantitative airflow metrics for voluntary throat clearings compared to voluntary and induced reflexive coughs in a healthy population.

We observed statistically significantly lower PEFR and lower CEV for throat clearings compared to voluntary coughs (*p* < 0.001) in a healthy population. The confidence intervals did not overlap and were very compact for both manoeuvres, suggesting consistency between participants. These aerodynamical differences are confirmed by anatomical and acoustical differences between both manoeuvres. A throat clearing may start without prior inspiration and requires partial vocal fold closure only (Laciuga et al. 2016; Xiao et al. 2014). The throat clearing is characterized by a continuous increase of the velocity of the adductive vocal fold motion just before the compression phase (Iwahashi et al. 2016). This increased velocity during the precompression adduction is considered to produce a high fold terminal velocity and high vocal fold collision force (Iwahashi et al. 2016). During the compression phase, the closure of the glottis is incomplete with a steady narrowing of the glottal space throughout the expiratory phase (Iwahashi et al. 2016; Vertigan and Gibson 2016). Acoustically, a throat clearing is characterized by one main fragment composed of stable acoustical oscillations and low frication noise all along the signal (Mootassim‐Billah et al. 2023). Visual observations of airflow waveforms show that throat clearings display a sequence of air pulses (low peak flows) instead of a single high one that would require a large inspiratory volume (Figure 1).

Conversely, a voluntary cough starts with a large inspiratory volume, followed by complete laryngeal closure, a forced expiratory effort, and a reopening of the glottis (Widdicombe and Fontana 2006). Acoustically, one observes a strong onset‐burst, followed by high frication noise and an optional voiced fragment (Mootassim‐Billah et al. 2023). Furthermore, compared to throat clearing, coughing involves other laryngeal structures in addition to the true vocal folds. Indeed, after closure of the true vocal folds, medialization of the false vocal folds occurs together with a closure of the supraglottic structures (contact between the petiole of the epiglottis with the arytenoids) (Iwahashi et al. 2016). This added laryngeal closure is considered necessary to prevent air from escaping during the compression phase of coughing (Iwahashi et al. 2016). Another physiological difference with throat clearing is that the true vocal fold motion during coughing is faster during expulsion abduction than during precompression adduction (Iwahashi et al. 2016). These combined aerodynamical, anatomical and acoustical differences between both manoeuvres suggest higher subglottic pressure and higher expired air volume to exist in voluntary coughs compared to voluntary throat clearings. The functional implications of these differences are that voluntary cough inefficiency is attributable to respiratory and/or laryngeal subsystem damage, whereas throat clearing inefficiency will mainly be caused by laryngeal subsystem damage.

However, in clinical practice, one observes that throat clearings are also spontaneously produced for clearance of pooling in the pharynx (valleculae and pyriform sinuses). Indeed, it is speculated that the variable contact between the tongue base and the pharyngeal wall during a throat clearing would facilitate residue evacuation. In addition, it is reported that pharyngeal mucosal inflammation with various underlying causes (e.g., laryngopharyngeal reflux, gastroesophageal reflux or asthma) may cause the apparition of chronic throat clearing, and conversely that the management of chronic throat clearing reduces inflammation of the pharyngeal mucosa (Acharya et al. 2007). This suggests that throat clearing production involves pharyngeal structures as well. Therefore, it is hypothesized that throat clearings might play a role in clearing pharyngeal pooling (pre‐swallow secretions and post‐swallow pharyngeal food residue). Given that pooling spillage is regarded as a clinical predictor of prandial penetration/aspiration in the lower respiratory tract (Farneti 2008; Murray et al. 1996; Neubauer et al. 2016), it would be of value to assess throat clearing objectively as a distinguishable protective manoeuvre to assess pharyngeal subsystem damage.

Voluntary coughs were also compared to induced reflexive coughs. The PEFR was lower for reflexive coughs compared to voluntary coughs. This confirms findings of numerous previous studies that investigated healthy subjects or patients (Brandimore et al. 2015; Curtis and Troche 2020; Mills et al. 2017; Wheeler Hegland et al. 2014). One explanation is that compared to voluntary coughs, the possible lack or reduction of the prior inspiration in reflexive coughs, lowers the expired volume (Smith et al. 2012). A second explanation is the simultaneous activation of accessory and respiratory muscles during a reflexive cough, without voluntary regulation (Lasserson et al. 2006). In contrast, these muscles are activated sequentially during voluntary coughs. This means that airflows can be voluntarily increased during voluntary cough, depending on the instruction (e.g., ‘cough hard’) or the need perceived (Lasserson et al. 2006). Furthermore, acoustic features describing voluntary and induced reflexive cough sounds also differ statistically significantly (Mootassim‐Billah et al. 2023). Compared to a voluntary cough sound, a reflexive cough sound presents a stronger convex curvature and steeper slope of the amplitude contour as well as a higher kurtosis and sample entropy, suggesting a very sudden release following laryngeal closure in reflexive coughs (Mootassim‐Billah et al. 2023). Physiologically, it is also observed that following reflexive cough expulsion, the true vocal folds close or do not close depending on the subject, presumably to further protect the airways (Britton et al. 2012). Acoustically, this is confirmed by visually observed higher amplitude and kurtosis values at the offset of the reflexive cough signals (Mootassim‐Billah et al. 2023). In summary, we may assume that the absence or reduction of a prior inspiration, the simultaneous and uncontrolled respiratory and accessory muscle activation, as well as the urgent need for airway protection in reflexive coughs (stronger laryngeal closure and reclosure after expulsion), contribute to lower expiratory airflows in reflexive compared to voluntary coughs.

Clinically speaking, throat clearing is the manoeuvre with the lowest PEFR. That is explained by a lacking/reduced prior inspiration and steady partial vocal fold closure throughout the expiratory phase. Because an effective throat clearing depends mainly on adequate narrowing of the glottis, this manoeuvre may help remove material at the laryngeal level via a sequence of low air pulses. These observations suggest that throat clearing could be useful for dysphagic patients at risk of penetration and showing impaired lung function. Nevertheless, it remains uncertain if a throat clearing alone is sufficient to effectively clear material from the lower airways. Also, it is hypothesized that the contact between the tongue root and the pharyngeal wall during a throat clearing may assist in clearing residue from the oropharynx and hypopharynx. In dysphagic patients at risk of pharyngeal pooling and subsequent airway spillage, an effective throat clearing throughout the meal might contribute to the clearance of pharyngeal residue.

Voluntary coughs display the highest PEFR because they require a large inspiratory volume followed by closure of the true vocal folds and supraglottic structures before a controlled forced air expulsion. Given that a voluntary cough is not preceded by a sensory component, it is not expected to inhibit penetration/aspiration. Nevertheless, it may contribute to the expulsion of unwanted material from the airway regardless of whether or not a reflex cough was triggered. Although no data were collected from individuals with dysphagia in the present study, it could be hypothesized that advising the use of voluntary coughs—either as isolated events or in short sequences—during meals or swallowing assessments might help facilitate the clearance of suspected or observed material located in the laryngeal vestibule, trachea, or lower airways.

Reflexive coughs display higher PEFR than throat clearings. This strongly suggests a lung volume contribution and a larger glottis opening during the expiratory phase compared to throat clearing. However, PEFR is lower in reflexive compared to voluntary cough because the former is produced with smaller lung volume and involves a marked laryngeal closure (and reclosure) after expulsion. Because the efficiency and the promptness of the latter depend on the integrity of peripheral sensory receptors, it is widely reported as the first defence against penetration/aspiration. In a dysphagic population at risk of sensory deterioration, reflexive cough should therefore be assessed during swallowing evaluation.

The hypotheses and interpretations proposed in this discussion remain speculative and should be viewed as preliminary, particularly in the absence of data from dysphagic patients. Further research involving individuals with dysphagia is needed to confirm the clinical relevance of these findings. Additional investigations incorporating instrumental measures—such as surface electromyography or imaging techniques—are planned to better understand the underlying mechanisms and potential functional impact of voluntary throat clearing.

Although PEFR and CEV offer valuable insights into the airflow characteristics of each manoeuvre, their validity as standalone proxies for airway protection or material clearance is limited. Therefore, any conclusions regarding the protective function of voluntary throat clearings, voluntary coughs, or reflexive coughs should be made cautiously, and ideally supported by direct evidence such as observed clearance of aspirated material or the prevention of airway invasion.

## 5 Limitations

Possible limitations of our study are the following. Asking the participants to produce both voluntary throat clearings and voluntary coughs without an agreed upon definition or distinction being available may have been confusing. In addition, providing a demonstration to participants who mixed up both manoeuvres may also have influenced the airflows produced. This observation confirms the auditory confusion reported in the literature between coughing and throat clearing (Curtis et al. 2024; Laciuga et al. 2016).

A second limitation is that participants were asked to produce single voluntary coughs and single voluntary throat clearings. This may have impacted manoeuvres and consequently airflow features. Besides, without this instruction, we could have compared CEV between voluntary throat clearing epochs, voluntary cough epochs and induced reflexive cough epochs. However, in the literature, the CEV has been compared for cough epochs with an unequal number of coughs (Borders and Troche 2022; Brandimore et al. 2018).

A third limitation is that the equipment used in this study has been initially developed to measure pulmonary function. The software does not report the predicted percentage of PEFR and CEV with regard to the cough function, taking into account the demographics and anthropometrics of the participants (e.g., age, gender, height, and weight).

A fourth limitation is that the influence of variables such as age, gender, height and weight was not explicitly taken into account in the manoeuvre comparisons of this exploratory study. One reason is that the ultimate focus of our overall research project was the intra‐participants’ correlations between aerodynamic and acoustic features. Also, the data collection was carried out during the Covid‐19 pandemic, which affected the inclusion of a representative sample of the healthy population. However, we retrospectively collected the anthropometric data of the majority of participants (35/40).

One additional constraint was the inspiration asked from participants prior to throat clearing. Indeed, literature reports that a throat clearing may be produced without prior inspiration (Xiao et al. 2014). The equipment used in this study required an inspiration prior to each manoeuvre to activate the airflow measurements. This instruction may have influenced the airflow features of the expiratory phase. However, one advantage of this constraint is that we analysed only one type of throat clearing.

## 6 Conclusion

This study reports comparisons of airflow features between voluntary throat clearings, voluntary coughs, and induced reflexive coughs in a healthy population. To our knowledge, this is the first study to report quantitative airflow data for voluntary throat clearings. Results show that voluntary throat clearings are aerodynamically significantly different from both voluntary and induced reflexive coughs.

## Funding

This study was funded by Association Jules Bordet.

## Ethics approval statement & participant consent statement

Data were recorded in the framework of a prospective interventional study that received ethical approval by the Jules Bordet Institute and the Universitair Ziekenhuis Antwerpen. This study is an ancillary analysis with a comparative purpose of data obtained from the same group of healthy volunteers that participated in our previous study on the acoustic analysis of voluntary coughs, voluntary throat clearings and induced reflexive coughs (Mootassim‐Billah et al. 2023).

## Consent

All participants provided written informed consent before being part of any study procedure. The investigators complied with the applicable regulatory requirements and adhered to GCP (Good Clinical Practice) and to the ethical principles laid down in the Declaration of Helsinki.

## Conflicts of Interest

The authors declare no conflicts of interest.

## Overall Research Project

This study is part of an overall exploratory research project retrospectively registered on ISRCTN (ID ISRCTN16540497) on 23 June 2023. The complete methodology of the overall research project was published in *Trials* (with SPIRIT checklist): https://doi.org/10.1186/s13063‐023‐07660‐y.

## Supporting information




**Supporting Information**:jlcd70160‐sup‐0001‐SuppMat.wav

**Supporting Information**:jlcd70160‐sup‐0002‐SuppMat.wav

**Supporting Information**:jlcd70160‐sup‐0003‐SuppMat.wav

## Data Availability

The datasets generated during the current study are not publicly available since they contain patient data and the informed consent does not include sharing‐data publicly. The measured outcomes (minima, maxima, medians, quartiles 1 and 3 as well as the bootstrapped confidence intervals) that we used for non‐parametric statistical analyses are made available in this manuscript.
